# Case Report of Dermoscopic Aspects and Reflectance Confocal Microscopy Description of Segmental Leiomyoma and Relative Management

**DOI:** 10.3390/medicina58121845

**Published:** 2022-12-15

**Authors:** Giovanni Paolino, Riccardo Pampena, Nathalie Rizzo, Matteo Riccardo Di Nicola, Santo Raffaele Mercuri

**Affiliations:** 1 Unit of Dermatology, IRCCS Ospedale San Raffaele, 20132 Milan, Italy; 2 Unit of Clinical Dermatology, Università Vita-Salute San Raffaele, 20132 Milan, Italy; 3 Surgical Pathology, IRCCS Ospedale San Raffaele, 20132 Milan, Italy

**Keywords:** dermoscopy, reflectance confocal microscopy, leiomyoma

## Abstract

Cutaneous leiomyoma is a benign tumor, mainly composed of smooth muscle cells and arising from the arrector pili muscle of hair follicles. The diagnosis of leiomyomas is of paramount importance, as they can often be associated with underlying malignancies (e.g., renal cell carcinoma, leiomyosarcoma) and specific genetic mutations. We report the case of a 27-year-old Caucasian male patient that presented to our attention with a rare segmental and Zoosteriform type II leiomyoma. We performed an analysis of the cutaneous lesions using dermoscopy, reflectance confocal microscopy (RCM) and histology. We found that, using dermoscopy, the leiomyomas showed a dermatofibroma-like appearance with a central hypopigmented area, peripheral delicate hyperpigmentation and also erythematous areas and ectatic vessels. RCM, although not specific, showed groups of hypo-reflective areas distributed in the most superficial papillary dermis, which in histology and immunohistochemistry corresponded to the most superficial protrusions in the papillary dermis of the tumoral bundles. Finally, we discuss the management of patients with multiple leiomyomas and stress the fact that, in the cases of multiple leiomyomas, an annual sonography of the kidneys associated with dermatological and (in women) gynecological consultations are needed to ensure the early identification of an underlying tumor. A genetic consultation to detect an eventual FH mutation is recommended, but since in some cases the FH result may be negative, the above recommended controls remain always of paramount importance.

## 1. Introduction

Cutaneous leiomyoma is a benign tumor, mainly composed of smooth muscle cells and arising from the arrector pili muscle of hair follicles [1]. Leiomyomas are usually divided into three main categories as follows: piloleiomyomas (derived from the erector pili muscle of hair follicles), angioleiomyomas (usually solitary lesions that originate from vascular wall smooth muscle tissue) and genital leiomyomas (scrotum or labium major, or from erectile muscle in the nipples). Among them, piloleiomyomas are the most common and present as pink–brown soft dermal papules or nodules that are distributed in single, clustered, segmental or disseminated patterns [2,3]. The skin accounts for 75% of all extra-uterine leiomyomas [1,2,3], and the most involved anatomic areas are the trunk and limbs. Cutaneous leiomyomas usually arise in adulthood during the fourth and fifth decades of life and may be sporadic or familial [2]. Considering that, in some cases, the diagnosis of leiomyomas can also imply specific and underlying genetic mutations (e.g., fumarate hydratase (FH) mutation in hereditary leiomyomatosis with renal cell carcinoma (RCC)), their rapid recognition by the clinicians is of primary importance [4]. Therefore, the synergistic contribution of different diagnostic methods (e.g., dermoscopy, reflectance confocal microscopy and histopathology) may improve the recognition of these cutaneous tumor lesions in order to start prompt treatment and follow-up.

## 2. Case Report

**Figure 1:**
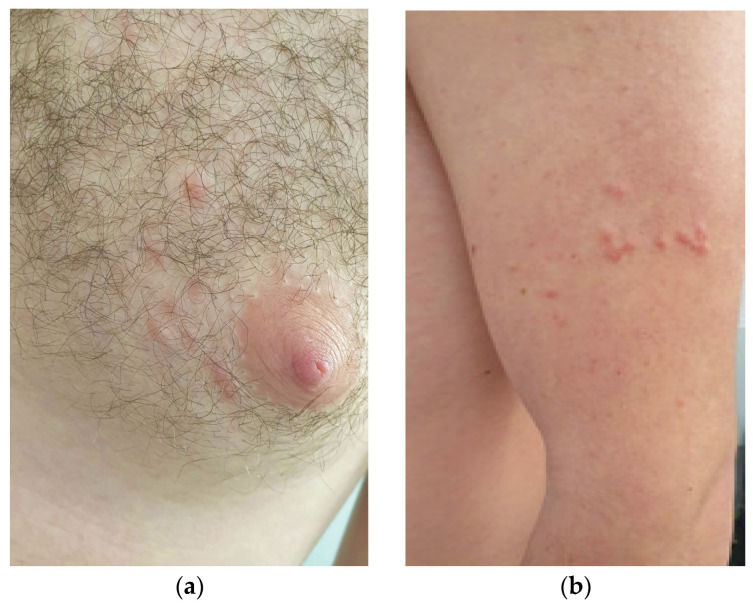
(**a**) Multiple, cutaneous, papulo-erythematous lesions, distributed in the left mammal areas; (**b**) the same patient with other lesions in the upper left limb. These lesions were painful and showed a general segmental (dermatomal-like) distribution.

A 27-year-old Caucasian male patient presented to our attention for a 2-year-old history of multiple, painful, scattered papulo-erythematous lesions, distributed at the left upper limb and left mammal areas in a segmental and dermatomal-like distribution (Figure 1a,b).

**Figure 2:**
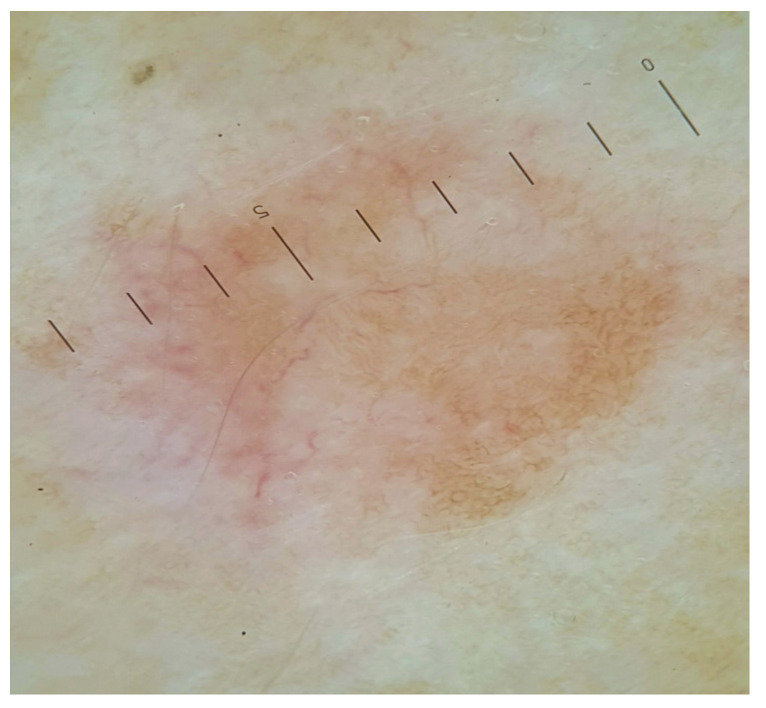
Dermoscopy showed a central hypopigmented area with a peripheral delicate hyperpigmentation and some erythematous arceiform areas with ectatic vessels.

His personal and familial medical histories were negative for cutaneous and systemic diseases. Dermoscopy of the lesions showed a central structureless and hypopigmented area with a mild peripheral pigmentation (dermatofibroma-like pattern), some peripheral arceiformes erythematous areas and scattered, ectatic linear vessels (Figure 2).

**Figure 3:**
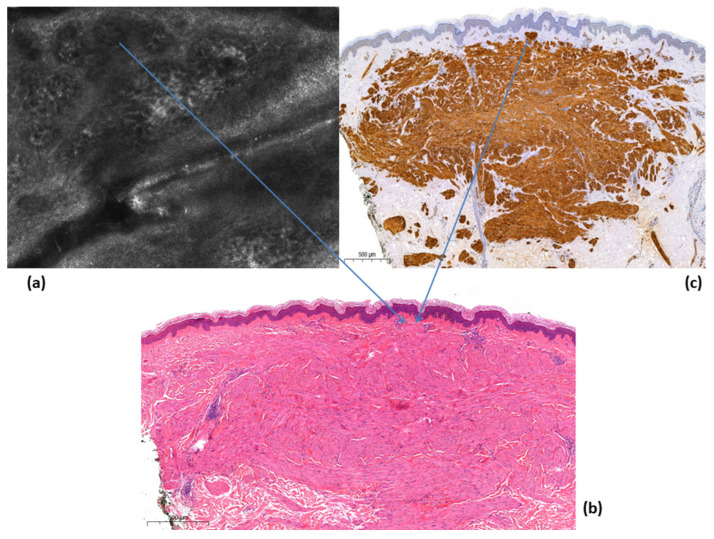
(**a**) Reflectance confocal microscopy (RCM) showed a general unspecific pattern with some hyporeflective structures in the upper papillary dermis and multiple ectatic vessels (accelerated blood flow); (**b**) the histology showed a leiomyoma, and the arrow indicates the superficial dermal propagation up to where the muscle bundles of the tumor reach, which corresponds to the multiple hyporeflective structures that were highlighted in RCM (Hematoxylin and Eosin, 50×); (**c**) the actine immunostain highlighted the bundles of the leiomyoma everted at the level of the superficial dermis that allowed the identification by RCM (Actine immunostain, 50×).

We decided to perform reflectance confocal microscopy (RCM) in three lesions before performing a cutaneous biopsy. RCM showed a general and unspecific alteration of the epidermis with aggregates of some hyporeflective areas, general disorder of the honeycomb pattern within the upper dermis, the presence of multiple and ectatic vessels (with accelerated blood flow) and some structures characterized by hypo-reflective aggregates that penetrated into the deeper dermis (not visible in RCM) (Figure 3a). The cutaneous histology showed a thin epidermis with a slight basal hyperpigmentation, while in the dermis, there were tangential and transversely sectioned circumscribed bundles of spindle cells with cigar-shaped nuclei without pleomorphism and without mitoses that were positive to actine and desmine (Figure 3b,c).

According to the clinic–pathologic correlation, a final diagnosis of segmental Zosteriform type II leiomyoma was rendered. The patient confirmed that no one in the family presented with these cutaneous lesions and/or systemic malignancies; a complete abdominal ultrasound was negative, and a medical genetic test (for the detection of a FH mutation) was requested.

## 3. Discussion

Cutaneous leiomyomas are single and/or multiple papular cutaneous lesions with a red–brownish appearance; when they are multiple, they usually arise in the trunk and extremities [1,2,3,4,5]. Cutaneous leiomyomas are typically painful, and the pain is exacerbated by cold, pressure or emotional stress [1]. Although they are considered benign lesions, leiomyomas can be associated with specific genetic abnormalities and malignancies; for this reason, their diagnosis is important.

Multiple piloleiomyomas usually occur in the age group of 10–30 years as in our case, presenting with a disseminated or grouped distribution. Grouped leiomyomas are fairly common; however, leiomyomas with a segmental and Zosteriform distribution are rare. Zosteriform leiomyomas can be categorized into two types as follows: (I) Type I is characterized by the presence of a dermatomal distribution; and (II) Type II is characterized by a segmental disposition, superimposed on scattered and isolated lesions. Our case showed a Zosteriform type II distribution because of the occurrence of the multiple clustered lesions in the left upper limb and left chest; in the literature, very few cases have been reported with this type of distribution [6,7,8].

In our case, dermoscopy revealed the presence of central hypopigmented areas with a peripheral delicate brownish pigmentation; these aspects were also observed by Di Luvio et al., where leiomyomas showed a dermatofibroma-like appearance [3]. However, contrariwise to Paschoal et al., we did not see hyperpigmented circular and/or elongated structures within the central hypopigmented areas [9]. In this regard, the delicate pigmentation that we observed in the leiomyomas is related histologically to a reactive epidermal basal hyperpigmentation that is usually present in leiomyomas (Figure 3b).

Reflectance confocal microscopy (RCM) is a non-invasive diagnostic method that allows the visualization of cutaneous lesions in vivo at a near histologic resolution, providing the visualization of nuclear and cellular morphology of the skin to a depth of approximately 150 to 200 μm depending on the anatomical site [10]. Therefore, RCM is a valid and useful technique for cutaneous lesions localized in the epidermis and superficial dermis; however, for deeper lesions, the method loses resolution. Leiomyomas, being deep cutaneous lesions, are difficult to evaluate with RCM. This justifies the fact that, in the literature, there are no RCM descriptions of leiomyomas because of the deep dermal location of the proliferation. In our lesions, however, we found some peculiar features that can be recognized as constituent elements (but not specific) of leiomyomas, such as ectatic superficial vessels with accelerated blood flow (corresponding in dermoscopy with the visible vessels and in histology with the superficial ectatic vessels in the papillary dermis (Figure 2)) and some hyporeflective structures in the upper dermis (Figure 3a) that, in histology, are related to some circumscribed bundles that arrive to the extreme superficial dermal portion (Figure 3b,c). The hyporeflective areas, visible with RCM in the epidermis, are mainly associated with the brownish color in dermoscopy and the relative reactive epidermal pigmentation in histopathology. Although it is important to specify that these elements are not specific for a recognition of leiomyomas, at the same time, the simultaneous use of different non-invasive diagnostic methods such as dermoscopy and RCM can individually offer single recognition elements to improve their diagnosis.

Although, to date, no official screening guidelines for patients with multiple leiomyomas are present as well as for patients with Hereditary leiomyomatosis and renal cell cancer (HLRCC) syndrome, Smit et al. listed practical criteria to ensure an appropriate diagnostic approach [3,11]. A genetic analysis (DNA analysis to test for FH mutations) should be requested in patients with familial and/or multiple cutaneous and/or uterine leiomyomatosis (MCUL). However, unfortunately, some mutations are not detectable via standard sequencing analysis. Therefore, patients with cutaneous and uterine multiple leiomyomas should receive annual renal sonography, gynecological examination and cutaneous examination in order to exclude the onset of a RCC or a uterine and/or cutaneous leiomyosarcoma, respectively, regardless of the result of the genetic test for FH.

To date, there is no definitive treatment for cutaneous leiomyomas. Surgical excision can be performed in single cutaneous lesions, while for multiple lesions, the treatment is more difficult; in these cases, nitrogen cryotherapy and carbon-dioxide laser (CO_2_ laser) are valid therapeutic options to excise the lesions, although not always with satisfactory results. Finally, for painful cutaneous lesions, nifedipine, phenoxybenzamine and gabapentin may be prescribed to reduce the pain.

## 4. Conclusions

The peculiar feature of our case was the atypical segmental distribution of the lesions, highlighting how this distribution may be present in clinical practice and, therefore, its recognition by clinicians is important. We confirmed dermatofibroma-like features as reproducible dermatoscopic clues for the diagnosis of leiomyomas. For the first time, we provided to the literature RCM images of a segmental leiomyoma showing how (although not specific) some RCM features may have clinic, dermatoscopy and pathologic counterparts, allowing to improve the diagnosis of these rare lesions. Finally, we stress the fact that in cases of multiple leiomyomas, an annual sonography of the kidneys associated with dermatological and (in women) gynecological consultations are needed to ensure the early identification of an underlying tumor. A genetic consultation to detect an eventual FH mutation is recommended, but since in some cases the FH result may be negative, the above recommended controls remain always of paramount importance.

## Data Availability

Data are available upon request to the corresponding author.
